# Ultrasound-Guided Regional Anesthesia by Emergency Physicians for Hip Fractures and Delirium: A Randomized Clinical Trial

**DOI:** 10.1001/jamanetworkopen.2025.49337

**Published:** 2025-12-15

**Authors:** Jacques S. Lee, Jordan Chenkin, Robert Simard, Tina Bhandari, Michael Y. Woo, Jeffery J. Perry, Debra Eagles, Charles Wong, Andrew D. McRae, Eddy Lang, Joseph Newbigging, Marco L. A. Sivilotti, Ian Chernoff, Bjug Borgundvaag, Shelley L. McLeod, Donald Melady, Alex Kiss, Marcel Émond

**Affiliations:** 1 Schwartz/Reisman Emergency Medicine Institute, Sinai Health, Toronto, Ontario, Canada; 2 University of Toronto, Toronto, Ontario, Canada; 3 Department of Emergency Service, Sunnybrook Health Sciences Center, Toronto, Ontario, Canada; 4 Axe Santé des Populations et Pratiques Optimales en Santé, Centre de Recherché du Centre Hospitalier Universitaire de Québec-Université Laval, Québec, Canada; 5 Departément de Medécine d’Urgence, Centre Hospitalier Universitaire de Québec-Université Laval, Québec, Canada; 6 Department of Emergency Medicine, The Ottawa Hospital, Ottawa, Ontario, Canada; 7 University of Ottawa, Ottawa, Ontario, Canada; 8 University of Calgary, Calgary, Alberta, Canada; 9 Queen’s University, Kingston, Ontario, Canada; 10 Department of Epidemiology and Biostatistics, Sunnybrook Research Institute, Toronto, Ontario, Canada

## Abstract

**Importance:**

Delirium complicates 20% to 62% of the estimated 1.5 million hip fractures annually. Meta-analyses suggest that point-of-care ultrasound-guided regional anesthesia (POCUS-GRA) may reduce delirium, but uptake by emergency department (ED) physicians is highly variable.

**Objectives:**

To measure the impact of a knowledge-to-practice intervention on uptake of POCUS-GRA by ED physicians, incidence and number of days of delirium, analgesic effectiveness, safety, and procedure time.

**Design, Setting, and Participants:**

This stepped-wedge cluster randomized clinical trial was conducted at 7 academic EDs from 4 provinces in Canada. The order in which ED physicians were trained at each site was randomized. ED physicians working at least 1 shift per week who did not regularly perform POCUS-GRA and patients aged 65 years and older with a hip fracture who were not delirious on arrival were included. Data were analyzed in August 2025.

**Intervention:**

A 2-hour structured training session on fascia iliaca blocks was followed by competency assessment, with a procedure bundle and email reminders provided.

**Main Outcome and Measures:**

Incidence of delirium within 7 days of ED presentation was measured using the Confusion Assessment Method, and duration of delirium was measured in days.

**Results:**

A total of 208 of 213 ED physicians (97.7%) who assessed 694 eligible patients with hip fracture (median [IQR] age, 81.0 [74.0-88.0] years; 483 female [69.6%]) were trained. Among these patients, 248 individuals were analyzed prior to training per intention-to-treat analysis, including 6 with protocol violations (control group), and 446 individuals after training (intervention group). The intervention increased blocks from 6 of 264 patients (2.2%) to 236 of 446 patients (52.9%) after training, for a change of 51.7%. After adjustment for a priori confounders, time, and clustering by site, delirium risk in the intervention group was reduced (odds ratio, 0.72; 95% CI, 0.57-0.93). Mean days of delirium was not reduced. Among 186 nerve blocks with pain effectiveness data, most nerve blocks were effective (107 nerve blocks [57.5%]with ≥50% pain reduction), quick (median [IQR] time, 15 [12-20] minutes; 113 nerve blocks [90.0%] <25 minutes), and safe (1 minor hematoma).

**Conclusion and Relevance:**

In this study, a knowledge-to-practice intervention improved nerve block uptake, and nerve blocks were quick, safe, and mostly effective. Despite suboptimal uptake, the study adds to the evidence that POCUS-GRA reduces delirium.

**Trial Registration:**

ClinicalTrials.gov Identifier: NCT02892968

## Key Points

**Question:**

Can a simple knowledge-to-practice intervention increase use of point-of-care ultrasound-guided regional anesthesia (POCUS-GRA) in the emergency department for older people with hip fractures, and does this reduce delirium?

**Findings:**

In this randomized clinical trial, a knowledge-to-practice intervention improved POCUS-GRA use, and nerve blocks were safe and quick. Despite suboptimal uptake and block effectiveness, odds of delirium were reduced in the intervention group.

**Meaning:**

In this study, nerve blocks provided by emergency department physicians after a feasible training program reduced delirium.

## Teaser

This randomized clinical trial investigates the effect of a knowledge-to-practice intervention on uptake of point-of-care ultrasound-guided regional anesthesia in older patients with hip fracture and on delirium incidence.

## Introduction

Delirium complicates 20% to 62% of the 1.5 million hip fractures occurring each year globally, doubling mortality, extending hospital stays by 7.8 days, and increasing nursing care burden, nursing home admissions, and risk of dementia.^1,2,3,4^ Delirium can also be life altering, leading to lasting psychological trauma to patients and their families.^5^ Mean costs are estimated at $43 669 annually and more than $65 billion globally.^6,7^

Previous meta-analyses^8,9,10^ found that regional anesthesia provided better pain management in hip fractures than parenteral opioid analgesics, and these studies provided moderate-quality evidence that regional anesthesia was associated with reduced delirium risk. Early administration in the emergency department (ED) is critical to provide rapid analgesia.^8,9,11,12,13^ Point-of-care ultrasound-guided regional anesthesia (POCUS-GRA) allows direct visualization of neurovascular structures and improves the safety and efficacy of regional anesthesia.^14,15,16,17^

Unfortunately, we showed that academic ED physicians rarely used any form of regional anesthesia for hip fractures (<5%).^18^ Inadequate training and perceived time to perform blocks were the most commonly identified barriers.^18^ Current population-based data showed that 18.5% of patients with hip fracture received a peripheral nerve block within 1 day of surgery.^19^ Thus, we developed a knowledge-to-practice (KTP) intervention to train and encourage ED physicians to perform POCUS-GRA.^20^

Our primary objective was to assess whether our KTP intervention increased the use of POCUS-GRA by ED physicians and whether this impacted the incidence and duration of delirium in older people with hip fractures. Secondary outcomes included efficacy, safety, and time to perform POCUS-GRA.

## Methods

The Research Ethics Board at all participating centers approved this randomized clinical trial and waived the requirement for patient informed consent. Instead, participating ED physicians explained the risks and benefits of the procedure to patients and obtained procedural consent as per their routine clinical practice. Research assistants subsequently sought informed consent from potential participants for administration of questionnaires and access to their medical records. We used the Adamis method to assess capacity.^21^ All participating ED physicians provided written informed consent, as did substitute decision-makers if a patient lacked capacity. Reporting of this trial follows the Consolidated Standards of Reporting Trials (CONSORT) extension for the reporting of cluster-randomized trials.

### Study Design

The ED Ultrasonographic Regional Anesthesia to Prevent Incident Delirium (EDU–RAPID) study was a pragmatic (designed to evaluate how the intervention worked in clinical conditions), multicenter, stepped-wedge, cluster randomized clinical trial^22^ (NCT02892968; see trial protocol in Supplement 1). Outcome assessors were blinded to the intervention status of patients. We chose this design^23^ because logistic challenges precluded simultaneous training of all physicians at a site, there was a high risk of contamination once physicians were trained, and randomly withholding effective pain relief (POCUS-GRA) represented a significant challenge to participant recruitment, as well as an ethical challenge for physicians. Instead, we randomized the order in which physicians were trained.

We enrolled patients with hip fractures and physicians at 7 academic EDs in 4 Canadian provinces: Rockyview, Kingston, Mount Sinai, and Ottawa Hospitals (Civic and General sites), plus Hôpital Enfant-Jésus and Hôpital Saint-François d’Assise. Sunnybrook Health Sciences Center piloted the trial and led training.^20^ We included emergency physicians working a minimum of 4 shifts per month who did not regularly perform POCUS-GRA (≤4 in the prior year). We included patients aged 65 years and older with a surgically treated hip fracture, excluding individuals with delirium on arrival, allergies to local anesthetic, anticoagulant use, minimal pain (≤3 of 10), or severe dementia (nonverbal or too impaired to complete interviews). We enrolled patients from June 2016 to January 2020.

### Description of KTP Intervention

We previously described our KTP intervention,^20^ which includes a 2-hour hands-on training session on the use of fascia iliaca blocks. ED physicians needed to successfully complete a competency-based checklist to complete training. Research assistants stocked the ED with POCUS-GRA equipment bundles containing all equipment needed to perform the procedure and sent email reminders to participating ED physicians.^20^

### Stepped-Wedge Cluster Design

The intended unit of cluster was the ED physician. The study biostatistician (A.K.) randomized the order of ED physicians training at each site by assigning a computer-generated rank to each physician. ED physicians with POCUS expertise were stratified (eg, if there were 4 experts at a site, 1 expert was assigned to each rank quartile). Patients treated by an ED physician prior to training were assigned to the control group. We used an intention to treat approach; even if an ED physician missed their randomized training session, their subsequent patients were assigned to the intervention group. Thus, each physician acted as their own control.

Training sessions were the sequence for the trial. Because of the complexities of scheduling busy ED physicians, the time between training sessions varied from approximately 4 to 12 weeks, and the number of ED physicians per session varied from 1 to 6.

### Physician Data Collection

Physicians were trained at study initiation to complete a data form^24,25^on all patients with hip fracture. They documented patient self-reported pain severity on a 10-point numeric rating scale at baseline. Once ED physicians completed their training, they also documented pain on the numeric rating scale at 30 minutes after POCUS GRA, any of a prespecified list of minor (ie, local hematomas) or serious (femoral artery or nerve puncture, hypotension, seizures, shortness of breath, anaphylaxis, or any other complication requiring treatment) complications,^24^ and the time required to complete the procedure. ED physicians also were trained to complete the Confusion Assessment Method, short form (CAM-S)^24,25^ on all patients with hip fracture to exclude patients arriving with delirium.

### Research Assistant Data Collection

All research assistants received standardized training for all study scales, including the Short-Blessed Test of mental status^26,27^; Mini-Cognitive Test,^28^ CAM,^24,25^ mobility status, Richmond Agitation Scale scores,^29^ and preinjury activities of daily living using the Older American Resources Scale.^27,30,31,32^ Research assistants reviewed standardized simulated delirium cases and had to demonstrate competence and reliability prior to independent assessments. All delirium outcomes were reviewed by the coordinating center to assure consistency across sites. Research assistants confirmed that all patients enrolled by ED physicians were delirium free on arrival by using the full version of the CAM-S supplemented with a validated electronic health record (EHR) review tool^33^ as recommended by the CAM-S training manual.^25^

Research assistants conducted daily follow-up CAM-S assessments and EHR reviews for the primary outcome, incident delirium. They also recorded the Mini-Cognitive Test^28^ and Richmond Agitation Scale daily until the patient was discharged from the hospital or the seventh postoperative day.

### Sample Size Calculation

Sample size was based on the primary outcome, the risk of incident delirium within 7 days. We expected each physician to treat 4 to 6 patients. Assuming 144 participating physician clusters and an intraclass correlation coefficient of 0.01 to 0.04, at the point at which half of the physicians have been trained, a sample size of 720 to 840 patients would provide 80% power with an α of .05 to detect an absolute difference in delirium risk of 7.6% to 8.0%. We stopped enrollment after we had screened 937 patients and enrolled 841 apparently eligible patients. However, 147 additional patients were found to be ineligible.

### Statistical Analysis

The intended unit of clustering was the physician, and the unit of analysis was individual participant (see trial protocol in Supplement 1). The primary outcome was the proportion of patients with incident delirium within 7 days of admission.

In 64 of 468 patients in the intervention group (15.5%), ED physicians treated 1 or fewer eligible patients during the study, precluding cluster analysis based on physician. Post hoc analysis revealed that the intraclass correlation coefficient was low (0.007) for the 134 clusters where 2 or more patients were assessed. Conversely, we observed significant variance in delirium rates across sites, ranging from 1 of 66 patients (1.5%) at site 3 to 39 of 95 patients (41.1%) at site 5, with an intraclass correlation coefficient between sites of 0.04. (Baseline cognitive impairment was also lower at site 3, occurring among 1 of 66 patients [1.5%], compared with 58 of 628 patients at all other sites [9.2%]). Accordingly, we accounted for clustering by site alone in our analysis using a generalized estimating equation model with a compound symmetry correlation structure, using a logit link function and a binomial distribution to adjust for the correlation among observations taken from the same site. Time or cohort effect were standardized across sites, and we controlled for these factors by including a variable defined as the time since the trial was initiated at each site in quartiles. We controlled for imbalances between groups that may exist despite randomization for the following important confounders identified a priori. Specifically, Inouye et al^34^ identified age, sex, time to surgery, baseline cognitive impairment, dehydration, and iatrogenic complications. These confounders have been extensively used in previous delirium research, including 1 prior randomized clinical trial of regional anesthesia.^12^ Our coprimary outcome was the duration of delirium in days; participants who became delirious on the operative day (day 0) had the maximum duration of delirium (8 days), and those who remained delirium free until the seventh postoperative day had zero days of delirium. We used a generalized estimating equation model with a log link function and a Poisson distribution for duration of delirium in days and reported the incidence rate ratio. The intervention was determined to be effective when 95% CIs for odds ratios did not cross 1. Data analysis was initially conducted in June 2022 and revised in August 2025 using SAS statistical software version 9.4 (SAS Institute).

Secondary outcomes included safety or complication rate, which was reported with 95% CIs. Median and IQR of time to block were reported. We measured the proportion of nerve blocks that could be considered effective based on existing literature^35^ and consensus among our study team as being a 50% relative reduction from the initial pain score at 30 minutes after the nerve block. We also collected data from the medical records on mean morphine milligram equivalents for opioids given in the ED.

## Results

Of 217 eligible physicians, 213 individuals (98.1% of those eligible; 67 female [31.5%]) agreed to participate, with a mean of 15.6 years of ED experience. We trained 208 of 213 participating ED physicians (97.7%), of which 197 individuals enrolled an eligible patient. Sites trained between 1 and 6 physicians per training session, and there were a total of 111 training sessions or sequences across all sites. Sites 1 to 5 began enrollment from June 2016 to February 2017, with sites 6 to 7 starting in January 2018. These large differences in start time and number of sequences required modification of the stepped wedge CONSORT diagram for legibility. The standard stepped wedge CONSORT diagram is in the Figure, and the modified version is in the eFigure in Supplement 2. The mean (weighted SD) number of physicians per training sessions was 1.8 (0.6) physicians. Once trained, 197 of 208 eligible physicians treated an eligible patient during the trial.

**Figure.**
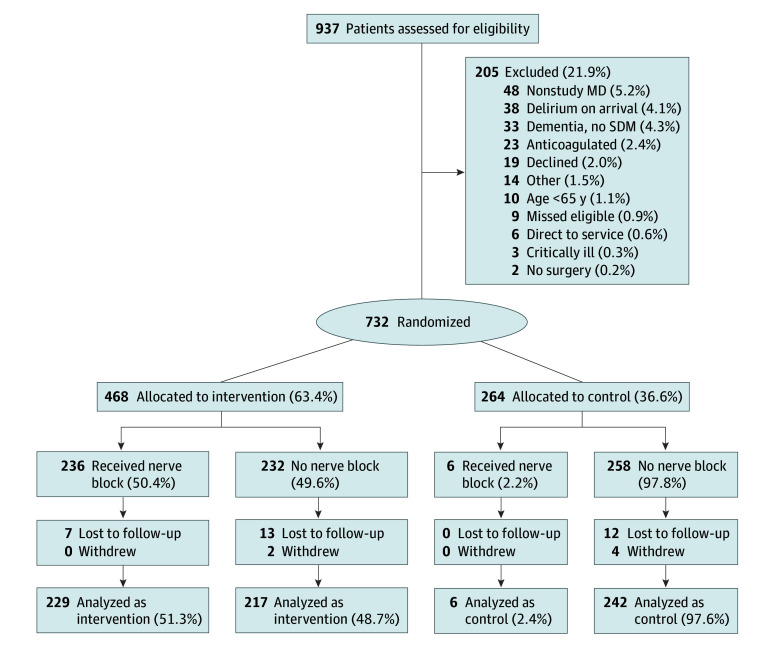
Study Flowchart MD indicates medical doctor; SDM, substitute decision maker.

We screened 937 patients with hip fractures, of whom 205 patients (21.9%) were excluded. Of 732 patients randomized, 264 individuals (36.6%) were treated before physician training and were assigned to the control group and 468 individuals (63.4%) were treated after training and were assigned to the intervention group. In the intervention group, 20 patients (4.2%) had missing outcome data and 2 patients (0.4%) withdrew. In the control group, 12 patients (4.5%) had missing outcome data and 4 patients (1.5%) withdrew. The remaining 694 participants (96.4% of the study sample; median [IQR] age, 81.0 [74.0-88.0] years; 483 female [69.6%]) were included in the analysis, including 248 patients (34.9%) in the control group per intention-to-treat analysis (6 with protocol violations) and 446 patients (64.3%) in the intervention group. Demographics of patients in control and intervention groups are provided in Table 1, and demographics of 193 patients with delirium (125 female [64.8%]; median [IQR] age, 85 [77.0-90.0] years) and 501 patients without delirium (358 female [71.5%]; median [IQR] age, 80 [73.0-87.0] years) are provided in Table 2. The number of physician per site varied from 12 to 51 physicians, and the number of participants enrolled per site varied from 24 to 202 patients. The mean (SD) number enrolled by each physician was 3.5 (5.1) patients and varied from 0 patients by 11 physicians to 22 patients by 1 physician (Table 3).

**Table 1.** Baseline Characteristics of Patients Analyzed in Intervention and Control Groups

Characteristic	Patients, No. (%)		
	Intervention group (n = 446 [64.3%])	Control group (n = 248 [34.7%])	All patients (N = 694 [100%])
Sex			
Female	300 (67.3)	183 (73.8)	483 (69.6)
Male	146 (32.7)	65 (26.2)	211 (30.4)
Age, median (IQR), y	81.0 (73.0-88.0)	82.0 (75.0-88.0)	81.0 (74.0-88.0)
No. missing data	0	0	0
Assessment scores, median (IQR)			
Short Blessed Test	5.0 (2.0-10.0)	6.0 (2.0- 10.0)	6.0 (2.0-10.0)
No. missing data	9	2	11
Pre–nerve block pain	6.0 (2.0-10.0)	5.0 (3.0-8.0)	6.0 (3.0-8.0)
No. missing data	172	63	235
Post–nerve block pain	2.0 (0.0-4.0)	4.0 (2.0-6.0)	2.0 (0.0-4.0)
No. missing data	262	246	508
Activities of daily living	14.0 (12.0-14.0)	13.0 (12.0-14.0)	13.0 (12.0-14.0)
No. missing data	4	5	8
Instrumental activities of daily living	13.0 (10.0-14.0)	13.0 (10.0-14.0)	13.0 (10.0-14.0)
No. missing data	4	8	12
Days to surgery, median (IQR)	1.0 (1.0-2.0)	1.0 (1.0-2.0)	1.0 (1.0-2.0)
No. missing data	0	0	0
No delirium risk factors	154 (34.5)	80 (32.3)	234 (33.7)
Dehydration (urea-creatinine ratio ≥0.1)	115 (27.8)	69 (27.8)	184 (26.5)
Cognitive impairment (Short Blessed Test score >4/28)	233 (52.2)	131 (52.8)	364 (52.5)
Iatrogenic complication	18 (4.0)	10 (4.0)	28 (4.0)
Delirium	116 (26.0)	77 (31.0)	193 (27.8)

**Table 2.** Baseline Characteristics of Patients by Delirium Status

Characteristic	Patients, No. (%)		
	No delirium (n = 501 [72.2%])	Developed delirium (n = 193 [27.8%])	All patients (N = 694 [100%])
Sex			
Female	358 (71.5)	125 (64.8%)	483 (69.6%)
Male	143 (28.5)	68 (35.2)	211 (30.4)
Age, median (IQR), y	80.0 (73.0-87.0)	85.0 (77.0-90.0)	81.0 (74.0-88.0)
No. missing data	0	0	1
Assessment scores, median (IQR)			
Short Blessed Test	4.0 (0.0-8.0)	10.0 (6.0-15.0)	6.0 (2.0-10.0)
No. missing data	3	4	11
Pre–nerve block pain	6.0 (3.0-8.0)	5.5 (3.0-8.0)	6.0 (3.0-8.0)
No. missing data	168	67	235
Post–nerve block pain	2.0 (0.0-4.0)	2.0 (0.0-5.0)	2.0 (0.0-4.0)
No. missing data	365	143	508
Activities of daily living	14.0 (13.0-14.0)	13.0 (11.0-14.0)	13.0 (12.0-14.0)
No. missing data	5	0	8
Instrumental activities of daily living	13.0 (11.0-14.0)	11.0 (7.0-13.0)	13.0 (10.0-14.0)
No. missing data	7	5	12
Days to surgery, median (IQR)	1.0 (1.0-2.0)	1.0 (1.0-2.0)	1.0 (1.0-2.0)
No. missing data	0	0	0
No delirium risk factors	205 (40.9)	29 (15.0)	234 (33.7)
Dehydration (urea-creatinine ratio ≥0.1)	133 (26.6)	51 (26.4)	184 (26.5)
Cognitive impairment (Short Blessed Test score >4/28)	212 (42.3)	152 (78.8)	364 (53.5)
Iatrogenic complication	18 (3.6)	10 (5.2)	28 (4.0)

**Table 3.** Patients, Training Sessions, Physicians, and Delirium at Each Site

Site	No.		All patients, No. (% of total)	Patients per physician, mean (SD) %	Patients, No. (row %)	
	Physicians	Training sessions			Developed delirium	No delirium
Site 1	11	8	24 (3.5)	2.2 (1.8)	6 (25.0)	18 (75.0)
Site 2	21	17	202 (29.1)	9.6 (4.4)	55 (27.2)	147 (72.8)
Site 3	26	12	66 (9.5)	2.5 (2.2)	1 (1.5)	65 (98.5)
Site 4	45	25	103 (14.8)	2.3 (1.5)	40 (38.8)	63 (61.2)
Site 5	45	27	95 (13.7)	2.1 (1.2)	39 (41.1)	56 (59.1)
Site 6	27	7	41 (5.9)	1.5 (1.0)	10 (24.4)	31 (75.6)
Site 7	22	15	163 (23.5)	7.4 (4.0)	42 25.8)	121 (74.2)
Total	197	111	694 (100)	3.5 (3.6)	193 (100)	501 (100)

Once trained, ED physicians completed nerve blocks on 236 participants (52.9%) in the intervention group (compared with 6 of 24 participants before the intervention [2.2%], a 51.7% improvement); there was 1 failed block attempt after training. Among 232 participants (49.5%) in the intervention group who did not receive a nerve block, reasons given included too busy (74 participants [32.0%]), difficult anatomy (38 participants [16.5%]), patient or caregiver refused the procedure (10 participants [4.3%]), physician forgot (9 participants [3.9%]), and other reason 4 participants [1.7%]), with no reason given in 96 participants (41.6%).

In the control group, there were 6 participants (2.4%) with crossover protocol violations, where a nerve block was completed in the control group. Typically, another physician who had already received training assisted the untrained treating physician for the patient with hip fracture prior to the scheduled training of the treating physician (Figure). There were an additional 3 failed attempts at performing a nerve block in participants in the control group. Thus, the failure rate among trained treating physicians was 1 of 237 attempts (0.4%) vs 3 of 9 attempts (33.3%) among untrained treating physicians.

Overall, 193 analyzed patients with hip fracture (27.8%) developed delirium, including 77 patients (31.0%) in the control group and 116 patients (26.0%) in the intervention group (Table 1). For our primary outcome, odds of delirium among patients in the treatment group were reduced (odds ratio, 0.72; 95% CI, 0.57-0.93). There was no reduction in the duration of delirium (incident rate ratio, 0.92; 95% CI, 0.67-1.25).

Of 235 nerve blocks completed in both groups, pain effectiveness data were available for 186 nerve blocks (78.8%). The mean (SD) pain score at 30 minutes after the block decreased by 3.1 points, from a baseline of 5.5 (2.7) of 10 points, and in 107 nerve blocks (57.5%), patients experienced a 50% or greater reduction in pain. The mean morphine milligram equivalent (MME) was lower in the intervention group (7.0 MME; 95% CI, 1.3-20 MME) compared with the control group (7.6 MME; 95% CI, 2.0-20 MME), although this was not significantly different given that the 95% CIs overlapped.

There were no serious adverse outcomes reported in the 236 nerve blocks performed and 1 minor complication, a hematoma at the injection site, that required no additional treatment (0.4%; 95% CI, 0.0%-2.4%). The time to perform nerve blocks was recorded in 126 blocks performed (53.4%), with a median (IQR) time to block of 15 (12-20) minutes, and 113 nerve blocks (90.0%) were completed within 25 minutes.

## Discussion

This pragmatic randomized clinical trial of 694 participants demonstrated that a KTP intervention reduced the odds of delirium (odds ratio, 0.72). By excluding physicians who routinely performed regional anesthesia, we confirmed the safety and effectiveness of our 2-hour training program among ED physicians with limited or no previous POCUS-GRA experience.

Our trial was inspired by 2 meta-analyses of regional anesthesia to reduce delirium.^36,37^ The authors concluded that there was moderate-quality evidence for reduced delirium. However, they included only 4 randomized clinical trials,^11,12,38,39^ and 1 trial contributed almost all cases of delirium (36 of 38 cases).^12^ Kim et al^40^ published a meta-analysis after our trial was initiated that added 7 randomized clinical trials with a total of 289 delirium cases. However, only 4 trials included preoperative regional anesthesia, and none provided nerve blocks in the ED. Overall delirium odds were reduced in patients randomized to receive a nerve block (odds ratio, 0.66; 95% CI, 0.36-1.22); however the 95% CIs crossed 1.0. To our knowledge, no trials to date have evaluated the effectiveness of translation into practice of regional anesthesia for reducing delirium in patients with hip fractures.

With 193 patients who had incident delirium, our trial adds to the evidence base supporting the effectiveness of early regional anesthesia in reducing delirium. We demonstrated the feasibility of training almost all ED physicians at multiple centers to perform safe and effective POCUS-GRA. One minor complication occurred among 236 nerve blocks performed, and 57.5% of nerve blocks were highly effective. Our KTP intervention improved nerve block uptake from 2.2% to 52.9%. Early regional anesthesia is now accepted as a Healthy Quality Ontario standard,^41^ and in 2023, the Australian and New Zealand Hip Fracture Registry reported that 87% of older patients with hip fracture in the registry received early regional anesthesia.^42^ Regional anesthesia is already known to provide optimal analgesia in hip fractures. The additional evidence provided by this study that regional anesthesia reduces incident delirium should challenge ED physicians and decision-makers to question whether POCUS-GRA should become the standard of care in their jurisdiction.

Future trials should confirm the impact of POCUS-GRA on delirium and explore the impact of improved early nerve block effectiveness on delirium risk. This may include training innovations to improve nerve block quality, duration, and uptake and exploring new approaches, such as pericapsular nerve group block and suprainguinal approaches.^43,44^ Finally, collaborations among the ED and anesthesia, orthopedic, and perioperative medicine departments are needed to improve nerve block rates.

### Limitations

This study has several limitations. A significant limitation of this pragmatic trial was that we analyzed 694 patients, which was less than our sample size of 720 patients (96.4%). We terminated the study after enrolling 841 apparently eligible patients. However, during data cleaning after we terminated trial enrollment, and prior to analysis, we discovered that 147 additional participants had exclusion criteria, had withdrawn, or had missing follow-up. COVID-19 restrictions prevented restarting the trial. Our post hoc power analysis demonstrated that with 694 analyzed participants, our trial was powered to exclude a reduction in delirium rates of 8.5% to 9.5%. Being underpowered is a conservative bias. Given that we demonstrated an odds reduction for incident delirium of 0.72, the impact of our premature termination appears to be limited. However, being underpowered may have contributed to the failure to demonstrate a reduction in delirium duration. In addition, our initial sample size adjusted for clustering but did not account for the stepped-wedge design.

In another limitation, we were unable to use physicians as the unit of analysis as originally intended because 13.7% of physicians treated 1 or fewer eligible participants. We adjusted for clustering by site instead of physician given the variability between sites in delirium incidence. The small number of sites is also a limitation. When randomization occurs by cluster, adjusting for a small number of clusters is essential.^45,46^ However, the situation is less clear when there is no cluster-level randomization.

Having 1 outlier site with a lower delirium rate (1.5%) than all other sites (range, 24.4%-41.1%) is another limitation. This may be due to a lower rate of contacting substitute decision-makers for patients with cognitive impairment at this site; 1.5% of enrolled patients had baseline cognitive impairment compared with 9.2% at all other sites. This site enrolled 9.5% of our sample; however, we cannot exclude the possibility of differential measurement of delirium outcomes at this site. Misclassification of delirium outcomes at this site would also bias results conservatively toward the null.

Even in expert hands, POCUS-GRA with femoral nerve blocks will not always be effective owing to variations in individual innervation anatomy and variability in fracture site (eg, intracapsular vs extracapsular sites). Nerve blocks in this study resulted in a greater than 50% reduction in pain for most patients; however, there was significant room for improvement in the effective nerve block rate. Again, this element of our pragmatic trial introduces a conservative bias. Improving the proportion of effective nerve blocks may have further reduced delirium. Another limitation is missing data for secondary outcomes collected by participating ED physicians, including the change in pain scores after POCUS-GRA and morphine equivalents.

## Conclusions

In this randomized clinical trial, widespread training in a multicenter setting of ED physicians with limited prior POCUS-GRA experience was feasible and resulted in effective, safe, and rapid analgesia for older people with hip fractures. Uptake of POCUS-GRA improved by 51.7%, and this was associated with decreased odds of delirium. Future research is needed to firmly establish the link between POCUS-GRA use and delirium reduction, identify strategies to improve POCUS-GRA uptake and efficacy by ED physicians, and assess the impact of policy and quality improvement initiatives on delirium in this large, at-risk population.

## Notes

Supplement 1.
Trial Protocol and Statistical Analysis Plan

Supplement 2. **eFigure.** CONSORT Modified Stepped Wedge Randomized Clinical Trial Flow Diagram

Supplement 3.
Data Sharing Statement
