# Supplementary material for: Ultrasound-Guided Regional Anesthesia by Emergency Physicians for Hip Fractures and Delirium: A Randomized Clinical Trial
Source: JAMA Netw Open. 2025 Dec 15;8(12):e2549337. doi: 10.1001/jamanetworkopen.2025.49337 (PMC12706686; doi:10.1001/jamanetworkopen.2025.49337)
Supplement: Supplement 1. — Trial Protocol and Statistical Analysis Plan [file jamanetwopen-e2549337-s001.pdf]

1.0 THE NEED FOR A TRIAL:

Overview

Hip fractures affect over 30,000 Canadians each year[1] and Canadians spend between $650 million and $1 billion dollars to treat these injuries yearly.[2] **Delirium**, or acute confusion, **complicates care in up to 62%** hip fracture patients.[3] Delirium doubles the risk of death and nursing home admissions, adds 7.8 days to hospital length of stay, and doubles the burden on nursing staff.[4-6] Daily care costs are 2.5 times higher for delirious patients.[7] A decade of research summarized in recent meta-analyses show that Regional Anesthesia (RA) provides better analgesia than the current standard of care, parenteral opioid analgesics.[8,9] **These meta-analyses also suggests that use of RA independently reduces the rate, severity and duration of delirium,**[8-11] but early intervention is critical.[12]

Unfortunately, uptake of regional anesthesia by Emergency Department (ED) physicians has been poor. We surveyed 100 ED physicians and found that **less than 5% routinely used any form of RA for hip fractures**. [13] The failure of widespread spontaneous uptake of this best practice is not surprising given the large body of research documenting that passive diffusion rarely changes practice.[14-24] The need for active strategies, informed by implementation science, is increasingly recognized. [14-24]

Providing high quality evidence that use of RA in ED hip fracture patients has important impacts on patient outcomes such as delirium is needed to improve uptake of this best practice. Not only will this influence individual emergency physicians' decisions to use RA in hip fracture patients, but it will lay the groundwork for guideline development and influence health policy decision makers.

The **ED** **U**ltrasonographic **R**egional **A**nesthesia to **P**revent **I**ncident **D**elirium (EDU –RAPID) study is a pragmatic cluster randomized clinical trial of a **knowledge-to-practice (KTP) strategy,** to train ED physicians to use ultrasound guidance to improve the ease and safety of a nerve block technique.[25,26] This trial will test whether our knowledge to practice intervention (see 1.3.7) ***reduces the rate of delirium through increased use of U/S guided regional anesthesia*** (USGRA) ***by ED physicians***.

1.1 Problem to Be Addressed.

Delirium complicating hip fractures is a major problem. As is further described below: 1) The Canadian health care system is currently challenged in caring for the heavy burden associated with hip fracture repairs (see 1.1.1) and this will worsen over the next 20 years (see 1.1.2); 2) Delirium complicates post-operative recovery in two-thirds of hip fracture patients (see 1.1.3); and 3) Post-operative delirium significantly impacts these patients outcomes and health care resource use (see 1.1.4).

1.1.1 Current Burden of Hip Fracture Care.

Currently, hip fractures affect over 30,000 Canadians each year.[1] With an average cost of $34,946 per patient, Canadians spend between **$650 million and $1 billion dollars** to treat these injuries yearly.[2] For patients who are unable to return home, the cost of care is twice as high.[2] Hip fractures **primarily affect older people - 87.6% occur among patients 65 years of age or older**, and the rate of hip fractures increases exponentially with age, more than quadrupling from age 65 to 85.[27,28] Women account for 75% of all hip fractures.[28,29]

1.1.2 Future Trends in Hip Fracture Rates.

The above data suggest that the Canadian health care system is currently heavily burdened by hip fractures. There is a significant risk that this current burden will increase over the next 20 years, due to the ageing of the historically large cohort of Canadians born after 1945 now surviving past age 65, [30] also known as the "Silver Tsunami".

Recent Canadian data showed that the total number of hip fractures has increased between 1985 and 2005 ***despite substantial reductions in the age-adjusted hip fracture rates***.[28] Similarly, the total number of hip fractures has ***increased*** in the US despite similar reductions in the age-adjusted rates.[28,31] One U.K. study projected that even accounting for a 9.6% reduction in the incidence of hip fractures, the total ***number*** of hip fractures will increase by at least 45%, and may increase by as much as 75%.[32] Thus, there is evidence that reduced age-adjusted hip fracture rates are not **keeping pace with the rapid increase in the total number of older citizens suffering from hip fractures.** All hip fracture patients require care in the ED, one of the most stressed segments in the health care system.

1.1.3 Hip Fractures and Delirium.

Unfortunately, delirium is a **common,**[33,34] **serious** [3,6,7,35-45] and potentially **preventable** [2, 26-30] complication of hip fractures. In a meta-analysis of 1,823 patients

from 12 trials, Bitsch et. al. found an average rate of post-operative delirium of 35%,[33] with rates as high as 62% reported. [34] Bruce et al.[34] published a systematic review and meta-analysis focused primarily on incident post-operative delirium. They excluded studies that did not use a validated measure of delirium and interventional studies. Their pooled delirium incidence estimates from 16 studies of 2203 older patients was 21.7%. Our pilot study found a post-operative delirium rate of 27/67 hip fracture patients (40.3%, 95% CI 28.5% to 53%, see "Summary of Progress" and section 1.3.9 for details).

1.1.4 **Impact of Delirium on Patient Outcomes** Delirium has been shown to negatively impact nearly every outcome studied. Many patients who suffer from delirium have distressing memories of the event; feel shame at their behavior, and some worry that they are losing their minds.[46,47] Family-members who witness delirium recount feeling disturbed and helpless.[46-48] In addition, delirium increases the risk for functional decline and slows recovery from surgery.[39,49-52]

A previously under-recognized consequence of delirium is the prolonged time to recovery of baseline cognitive function.[38] The average time to return to baseline cognition was 6 months, and 41% of hip fracture patients with post-operative delirium had ongoing cognitive deficits at 12 months.[41,53-55] A quarter of patients with delirium either die or are indefinitely admitted to a nursing home.[40,43,56] Finally, a number of studies have shown that delirium is an independent factor that doubles the risk of mortality even when the underlying medical diagnoses are controlled for.[6,35,45,57-59] Clearly, delirium is a dreaded complication of hip fractures for individual patients.

1.1.5 Impact of Delirium on the Health Care System Given the numerous well documented negative impacts associated with delirium, and the fact that delirious patients are unable to cooperatively participate in their recovery,[60] it is not surprising that delirium adds 7.8 days to each hospital length of stay,[5] doubles the risk of nursing home admission,[8,43,53,61] and increases the cost and length of nursing home admissions.[44] The burden on nursing staff caring for delirious patients is doubled, [4] and daily care costs are 2.5 times higher for delirious patients.[7]

Delirium is an expensive complication of medical care. In the USA, delirium costs $143 - $152 billion annually.[4,7] No comparable Canadian cost data were available. These personal and fiscal costs are all the more troubling as evidence from several meta-analyses suggests that post-operative delirium ***can be prevented*** by using regional anesthesia.[8,9,33]

1.1.6 Effectiveness of Regional Anesthesia to Prevent Delirium in Hip Fractures

Abou-Setta et al. published a systematic literature review and meta-analysis on the effectiveness of different pain management techniques for hip fractures. They found 4 randomized clinical trials [10,11,62,63] (n=461) and two cohort studies [64,65] (n = 634) that reported the impact of RA on delirium in hip fractures. In one trial from Hong Kong and a second from Argentina, ED physicians administered the RA. In the third trial based in Greece, Orthopedic surgeons administered the block once patients had arrived on the ward, and in the fourth trial, RA was administered by an anesthetist pre-operatively. In the meta-analysis, a significant reduction in delirium was found, with an odds ratio (OR) from the four RCTs of 0.36, (95% CI 0.17 to 0.74), and an OR for the two cohort studies of 0.24 (95% CI, 0.08 to 0.72). The authors concluded that there was moderate evidence that regional anesthesia was effective for managing acute pain, using the AHRQ modification of the Grading of Recommendations Assessment, Development and Evaluation (GRADE) framework,[66] corresponding to moderate confidence that the evidence reflects the true effect, although further evidence may change confidence in the estimate(see Appendix 15 for details). They also concluded that perioperative use of RA independently reduces the severity and duration of delirium[8,10,11] and that pre-operative administration was more effective than post-operative.

1.1.7 Potential Mechanism for Regional Anesthesia to Prevent Delirium Proposed mechanisms by which RA may prevent delirium include improved pain control[62], minimizing the use of narcotics[67], minimizing sedation [62], or the faster onset of pain control.[12] One randomized trial found that hip-fracture patients who were given a nerve block were less heavily sedated and had lower delirium rates.[68]

1.2 Principal Research Question:

The goal of the EDU-RAPID study is to test whether a **knowledge to practice intervention** to train,

facilitate and encourage ED physicians to use ultrasound-guided regional anesthesia **can reduce new cases of delirium following hip fractures.**

1.2.1 Primary Objective: To measure the proportion of patients with **incident delirium** in the first 7 days post-injury among patients treated by ED physicians randomized to the KTP intervention group compared to patients treated by control group ED physicians. We will also measure time to onset of new delirium, up to 7 days post-injury.

1.2.2 Secondary Objectives: Our secondary objectives include measuring the impact of the KTP intervention on: 1) The rate of regional anesthesia use by intervention physicians for hip fracture patients; 2) Time to perform the block; 3) Pain Severity 30 minutes after administration of regional anesthesia; 4) Effectiveness of block at 30 minutes; 5) Supplementary narcotic analgesics used pre-operatively, measured in morphine equivalent units; 6) Maximum severity of delirium using the validated Delirium Index (DI);[48] 7) Time of onset to delirium; 8) Cognitive status at hospital discharge; 9) Complications from the regional anesthesia including hematoma and persistent nervous dysfunction; 10) Adverse events, including in-hospital falls, cardiovascular events and deaths; 11) Hospital length of stay; 12) Functional status before hip fracture,at one month and one year follow-up; 13) Proportion of patients who return to independent living at discharge; and 14) Mortality within 1 year post-injury. For more details on measurement, please see **2.8.2 below** and **2.16** for analysis.

1.3 Why Our Proposed Trial is Needed Now?

1.3.1 Rising Pressures Placed On The Health Care System by Delirium and Hip Fractures

As outlined above, hip fracture is a common injury that is frequently complicated by delirium. Both conditions occur predominantly in the rapidly growing population of older adults. This is of particular concern given evidence that the health care system is currently challenged to provide timely access for hip fracture repair. In 2005, all Provinces and Territories adopted a set of evidence-based benchmarks. [69] For hip fractures, surgical repair within 48 hours was chosen as the national quality benchmark, since delays beyond 48 hours are associated with increased mortality, disability, and increased delirium.[70-72] Despite this, the latest national population-based data from the Canadian Institutes for Health Information (CIHI) shows that only 62.7% of Canadians had their hip fractures repaired within 48 hours, while only 58.8% of patients in the central Toronto achieved this benchmark - 16% waited longer than 72 hours for surgery.[73] When time spent waiting in the ED prior to admission is included in the analysis, only 47.3% of patients in Toronto had surgical repair within 48 hours.[74]

The fact that such a significant proportion of hip fracture patients face long delays has two important consequences. First, patients waiting experience significant pain and this increases the difficulty of caring for hip fracture patients.[75] Further, prolonged stays in the ED are known to double the risk for developing delirium.[76,77] One study showed a 5% increase in delirium for every hour waiting for surgery. It is now well established that patient's length of stay is a major determinant of hospital crowding.[78-80] Because delirium increases hospital length of stay and increases the number of patients needing nursing homes after their hip surgery,[5,28,35,39,81] delirium may contribute to hospital crowding. Delirium has the potential to worsen delays to surgery, in turn, increasing delirium rates. Thus, it is critical to test potentially effective interventions such as RA in hip fractures that may reduce delirium now to avoid this vicious cycle, and reduce the current and future strain that delirium complicating hip fractures places on our health care system.

1.3.2 Need for an Emergency Department based trial Because *almost all hip fracture* ***patients are initially assessed in the ED***, can arrive at any time of day, and often spend extended time periods in the ED,[73,74] we believe that ED physicians are best placed to provide RA to ensure that the largest number of hip fracture patients will have access to this best-practice. Not all hospitals have an in-house anesthesiologist around the clock, thus many institutions would be challenged to have anesthesiologists provide regional anesthesia in the ED. In one retrospective Australian study set in a pediatric trauma ED, the mean waiting time until administration of the femoral nerve block was 75± 9.6 minutes for ED physicians compared to 124± 14.2 minutes for anesthesia or orthopedic consultants.[82]

1.3.3 Suitability of Regional Anesthesia for use in the ED There are 7 studies examining the

use of regional anesthesia by ED physicians for hip fractures, with three using an ultrasound-guided technique.[12,25,63,83-85] In no study was a failure to learn the technique reported, likely due to the fact that the procedure is intrinsically simple and fast to perform (see 2.2.1 below). A demonstration of the technique is available at http://www.youtube.com/watch?v=C08whkeYzPk. Fletcher et al. trained all 14 emergency physicians at a mid-sized British ED to perform the 3 in 1 femoral nerve block using surface anatomy landmarks during a 30 minute instructional session followed by supervised practice on a mannequin.[12] The authors comment that all physicians were able to achieve competency. Graham reported block completion in under 10 minutes in 40 patients.[63] Our co-applicants (MW, JC) have extensive experience teaching ultrasound guided modified femoral nerve block, and have pilot tested the effectiveness of our training on a group of 13 residents in Toronto and 11 in Ottawa, none of whom work at participating hospitals. All 24 participants were able to successfully perform U/S guided regional anesthesia after 2 hours of training using an anatomically accurate ultrasound mannequin (see 2.21 and Appendix 1). Participants gave the training program an average evaluation score of 4.8/5.0.

1.3.4 Innovative use of Ultrasound to Guide Regional Anesthesia An innovative aspect of the proposed trial is the use of ultrasound to guide placement of the local anesthetic. Use of ultrasound has been shown to improve the success rate and reduce complications compared to traditional external landmarks techniques, without prolonging the time to perform the procedure.[25,26] This improved success and safety is believed to be due to the direct visualization of the position of anesthetic being instilled relative to the nerve and vessels**. No previous study on the effectiveness of regional anesthesia in the ED to prevent delirium has used ultrasound guidance**.[8,9,33]

1.3.5 Time is Ripe to Study Ultrasound Guided Regional Anesthesia in Hip Fractures

As evidence supporting the importance of ultrasound in the ED has increased over the last decade,[56-65] there has been a rapid expansion in its use.[66-70] As a result, the Royal College of Physicians and Surgeons have made basic ultrasound competency mandatory for all Emergency physicians graduating after 2010. Ultrasound equipment is thus required by any institution that trains emergency medicine residents. One of our collaborators (MW) conducted a national survey of Canadian emergency medicine training sites and showed that 82.4% performed ultrasound in their ED.[70] A US survey revealed that 92% of centers had ED ultrasound.[69] We surveyed 100 ED physicians at 3 academic centers (response rate 74%).[13] Although 89% stated that they supported routine use of regional anesthesia in hip fractures in the ED only 5% currently administered regional anesthesia "almost always". A lack of training was identified as the principle barrier to use of this technique by 57%.

1.3.6 Why Is Another Trial of Regional Anesthesia Needed? Evidence from recent meta-analyses provide "moderate" GRADE[66] level evidence that USGRA reduces post-operative delirium [8,9,33] but problems exist with the existing studies. First, of the four existing trials, delirium was the primary outcome in only one trial of 207 patients who received a block from an Orthopedic Surgeon.[11] Regional anaesthesia was not administered in the ED, nor was ultrasound guidance used. All four trials were published in journals not routinely read by ED physicians. Only 18% of physicians who completed our survey felt that there was a high level of evidence that regional anesthesia reduces delirium in hip fractures. The fact that ED physicians are not convinced that U/S guided regional anesthesia will reduce delirium.[13] suggests the need for a definitive trial (see appendix 15). We also conducted a survey that showed that 90% of responding ED physicians would increase the priority given to USGRA in hip fracture patients if evidence from a RCT demonstrated an improvement in delirium outcomes (see 1.3.9). Thus this trial is needed to help change practice and improve the care of older Canadians. Such high-quality evidence of impact on delirium would allow our well positioned team to transition USGRA from a best practice to the standard of care, improving the care of older Canadians.

1.3.7 Need for a KTP Strategy to Promote Uptake of Regional Anesthesia

In a perfect world, our proposed trial would not be needed. The evidence from meta-analyses that regional anesthesia is optimum for pain control [8,9,33] would be enough to lead to wide spread uptake. Unfortunately, uptake continues to be poor with only 5% of ED physicians are consistently using RA in hip fractures. Numerous systematic reviews have concluded that passive knowledge diffusion does not

change physician practice.[17,20-24,86-89] The simple existence of evidence that an intervention has some advantages does not mean clinicians will adopt it.[21,89]

We conducted a survey of 100 ED physicians to understand barriers to uptake. While 89% of respondents were in favor of the use of RA, only 31% rating use of RA as very or extremely important.[13] ED physicians must constantly prioritize which aspects of care are most important, and the timeliness of analgesic care specifically for older hip fracture patients is known to suffer during ED crowding.[90,91] Our survey also showed that physicians gave higher priority to the acute medical management of hip fracture patients.[13] Thus the current perceived "relative advantage" [92-94]of use of RA compared to standard opioid analgesics does not result in high prioritization of use of RA in these patients. This may explain why even existing financial incentives alone have not lead to significant uptake of use of RA.[95] We conducted a subsequent survey to probe methods of increasing ED physicians' prioritization of USGRA and found that 90% of ED physicians would increase the priority they give to this procedure if evidence existed that use of RA reduced the incidence of delirium (see Summary of Progress and 1.3.9).

Our KTP interventions build on our extensive pilot work, utilize strategies that have had the most success in changing physician practice [17,20,22-24,86-88] [15,16,88,96-99] (e.g. opinion leaders, coaching, reminders, feedback and demonstrated 83.3% uptake by physicians in our pilot study.)[100]

1.3.8 Justification for the Design of the Proposed Study Our proposed trial will be the largest randomized clinical trial to date, will be the first to use ultrasound guidance, and will be the first to initiate regional anesthesia at the earliest opportunity for all patients (i.e. in the ED). Our trial builds on previous literature demonstrating the ability of ED physicians to learn this procedure, as well as our own pilot work demonstrating the feasibility of implementing our KTP intervention locally among randomly selected physicians. Because our KTP intervention is clearly defined (see appendix 1) and requires modest additional resources, it will be easily generalized.

In traditional cluster randomized trials, randomization and clustering occurs at the level of institutions, but such trials ideally require a minimum of 10-12 institutions and are intrinsically expensive.[101-103] With smaller numbers of institutions, inter-institutional imbalances in outcome rates can bias the trial results of the trial.[104] Attempts to balance inter-institutional outcome rate differences using matched-pair designs may fail, as it may be difficult to assemble a well-balanced group of institutions, or the institutional attributes that impact the outcome may be unknown.[101,105]

Thus, we propose a **novel cluster design** where randomization and clustering occurs **at the level of the ED physician**, rather than by institution. Our proposed design is more efficient than if institutions are used as the unit of cluster and randomization, requiring half the numbers of sites and study coordinators that drive the cost of our trial. Our pilot study also revealed that training large groups of physicians at the beginning of the study would be impractical due to scheduling difficulties and the number of U/S machines and training staff available. The use of a **stepped wedged design** [106] permits the training of all participating physicians in small groups of 4, addressing both practical and ethical issues.

1.3.9 Pilot Work: Feasibility of the EDU-RAPID Study design To test the feasibility of our study design, we conducted extensive pilot work in the past 18 months. Survey#1) We surveyed 100 ED physicians[13] and found that 70% had never used RA in hip fractures, 26% used RA "sometimes or seldom", and only 5% used RA "almost always". Among the 30% of respondents that had ever used RA, 54% were only "somewhat or moderately" confident that they could provide a safe and effective block. The major barrier, a lack of training, was identified by 57% of respondents. Of the 26 respondents who had previously been trained in RA in the past, 63% learned an U/S guided approach, but only 27% were satisfied with their training as the majority learn the procedure during a two day course where RA for hip fractures is taught in 30 minutes as one of 12 other procedures. Survey #2: We then surveyed 140 ED physicians from Ottawa, Toronto and Quebec to assess their willingness to participate in our trial. The response rate was 84.3% and **92%**of the response stated they would participate, even though they could not choose their group assignment, and would agree to only treat patients according to their group assignment. It was clearly explained that half of participants had to wait up to 18 months before

receiving training. Survey#3: We also asked emergency physicians whether evidence from a randomized trial that showed a reduction in delirium among hip fracture patients receiving nerve blocks in the ED would change the priority they assign to performing nerve blocks and 90% stated it would or would definitely increase the priority given this procedure. Pilot Training#1) Next we tested the feasibility and effectiveness of our training program on 24 residents from Ottawa and Toronto (see 1.3.3 above for details).Pilot Training #2) Next 3 ED physicians at Sunnybrook were trained in USGRA to further test our educational materials, training, block kits, and data collection procedures prospectively test our patient enrollment and data collection strategy. Prospective Pilot Study - Single Site Randomized: We were then awarded $100,000 by the Ontario Ministry of Health Innovation Fund allowing us to fully implement our proposed full study protocol at one site, Sunnybrook. Four of 30 eligible physicians declined to participate (13.3%). We then *randomly selected* 13 of 26 eligible consenting ED physicians to receive training. Over 14 months, 206 hip fracture patients presented, 86/206 (41.7%) were ineligible (language barrier (14.1)%, age < 65 years (9.2%), severe dementia (9.2%), other (8.6%)). Some 25 patients declined to participate (12.1%), leaving 67 eligible patients. Of these, 24 were seen by one of 13 trained intervention physicians, and 20/24 received the block (83.3%, 95% CI 62 – 95%). The remaining 43 patients were enrolled as control subjects and received standard analgesia from the control ED physician. In-person interviews and telephone follow-up calls were completed for 66/67 enrolled subjects. Next, we used focus groups, chart reviews and live interviews of all eligible hip fracture patients seen during this 14 month pilot to intensively monitor for "cross-over contamination" or use of U/S guided RA by ED physicians not trained during our prospective pilot study. Only a single case of cross-over contaminations was observed when a nerve block was performed by an off service fellow. Note that no intervention physician trained any participants in the control group.

Our pilot study of 67 patients showed a delirium rate of 20/43 or **46.5%** (95% CI, 31.2 to 62.3%) among patients treated by the 13 control physicians and only 7/24 or **29.2%** (95% CI 12.6 to 51.1%) among patients treated by an intervention MD. While this promising data provides support for the need of our trial, given the lack of power, it does establish the effectiveness of our proposed intervention. **In summary**, our pilot work demonstrated no spontaneous uptake of USGRA for hip fractures, even though ED physicians favour the use of USGRA in hip fractures, 92.4% agreed to participate in our proposed cluster randomized trial, and 86.7% actually underwent randomization. We observed no evidence of cross-over contamination during a 14 months pilot study. We also successfully demonstrated the effectiveness of our training procedure and our KTP strategy in a group of randomly selected physicians, with 86.7% compliance. We have confirmed the feasibility of all of our data collection strategy and study procedures. Finally, our pilot data provides early evidence of a potential impact of USGRA on delirium, although this single-site data must be interpreted with caution.

1.4 Review of Existing Literature & Systematic Reviews: Our study rationale is supported by three systematic reviews. Abou-Setta et al. published a peer-reviewed publications[9] and a 278 page report providing a high level of detail on the evidence base for the effectiveness of pain management strategies in hip fractures.[8] From 93,567 citations, 83 unique studies were identified; including 64 randomized clinical trials, 5 non-randomized trials and 14 cohort studies. Of these, 32 were focused on regional anesthesia. Four randomized clinical trials[10,11,63,107] (n=461) and two cohort studies[52,108] (n = 634) reported the impact of regional anesthesia on delirium, although delirium was a primary outcome in only one trial.[11] The authors concluded that regional anesthesia was associated with a significant reduction in delirium, with an odds ratio (OR) from the four RCT's of 0.36, (95% CI 0.17 to 0.74), and an OR for the two cohort studies of 0.24 (95% CI, 0.08 to 0.72). The strength of evidence was rated "moderate" by GRADE criteria.[66] In addition, our sample size calculation is supported by two systematic reviews and meta-analysis that report pooled estimates of the incidence of post-operative delirium rate.[33,34] We used the estimated delirium incidence from Bruce et al given the additional methodological rigour and more conservative estimate of a 21.7% incidence rate (see 2.11 below).[34]

1.5 **How will Trial Results be Used** Demonstrating a link between our KTP

intervention,
increased USGRA uptake, the prevention of delirium, and reduced hospital length of stay, or reduced functional decline will provide powerful evidence that use of regional anesthesia for hip fractures **should be the standard of care in the ED**, and we will actively engage professional, institutional, regional and provincial decision makers to make that happen. Specifically, we will present our findings to the Council of Academic Hospitals of Ontario and have it rolled out at all academic hospitals in Ontario. In addition, our results will be presented at the Ministère de la Santé et des services sociaux du Québec, section aînés and Emergency physicians associations. As the lead of The Canadian Emergency Team initiative in aging,[109] Dr. Émond is in close association with Quebec provincial decision-makers, and knowledge users in geriatric/emergency/rehabilitation care. This group of 24 researchers will leverage the dissemination of our results nationally and internationally. Finally, provincial decision makers will be briefed on the potential benefits of wide spread implementation of our KTP intervention.

1.6 <u>**Risk to Participants and Ethical Issues**</u> REB approval had been granted at Sunnybrook and
L'Enfant Jesus (see Appendix 2). Eligible ED physicians will be asked for informed consent prior to randomization. We propose a stepped-wedged trial design [106] (see 2.1 below and Appendix 5) such that all physicians will be trained to use the nerve block, but the order of training will be randomized. Currently only 5% of physicians currently use the best practice of RA in hip fractures, and parental opioids is the current standard of care (see Appendix 17). As our trial is testing the effectiveness of a KTP intervention to teach ED physicians to use a procedure with a well established effectiveness and safety record, Sunnybrook and L'Enfant Jesus REBs ***have waived the requirement for patient informed consent prior to use of U/S guided RA***. Instead, participating ED physicians will explain the risks and benefits of the procedure to patients and obtain standard <u>clinical procedural consent</u> prior to administering USGRA. Trained Research Coordinators will subsequently seek informed consent from hip fracture patients for administration of study-related questionnaires and access to medical records (Please see Appendix 3 for the ED physician informed consent form and Appendix 4 for the patient informed consent form). All identifying information on study forms will be removed to protect privacy.

2.0 PROPOSED TRIAL

2.1 <u>**General Study Design:**</u> For both ethical and pragmatic reasons, (see p11 "Response to Comments") we propose a pragmatic, **multi-center cluster randomized stepped wedge clinical trial** [106]. A stepped wedged study is a type of one way cross-over study in which all participants start in the control group. During the course of the study, all participants cross over to the intervention group at regular intervals. Importantly, the order of training is randomly determined (see Appendix 5).

2.2 <u>**Planned Intervention and Control Groups**</u> All participating physicians will be assigned to a
cluster or group of 4-6 physicians. Three or four groups of Physicians will be trained across the 6 sites at 6-8 week intervals, allowing for training of all participating MD's within 72 – 78 weeks or 18 to 20 months. Order of training will be randomly determined, stratified by center. Any hip fracture patients seen by a physician who have not yet been trained during the enrollment period will serve as control subjects, and patients seen by a trained physician will serve as intervention subjects.

2.2.1 <u>Intervention Group:</u> We will use a proven knowledge to practice intervention that was designed by our KTP expert (DR) and ultrasound lead (JC) to incorporate current evidence on the most effective way to introduce a new procedural skill to a group of ED physicians. We have utilized this process and demonstrated 80% uptake of the intervention by trained physicians in our single-site pilot study. Required resources have been clearly defined so that it can be easily replicated in other ED settings .The following KTP strategies will be used to promote use of U/S guided regional anesthesia:
1. Opinion Leaders. We have identified teams of formal and informal opinion leaders among the ED Physician at each participating site (See Appendix 14). The ED Chiefs and/or the site investigator were

approached as formal opinion leaders (see letters of support). These formal opinion leaders were then asked to identify more informal opinion leaders to whom others would turn for advice regarding use of ultrasound. Both groups of opinion leaders have agreed to provide support during formal training as well in more informal circumstances in which their advice is sought regarding the trial.
2. Small Group Academic Detailing and Training. ED physicians will all receive a **2 hour training program** in groups of 4 by the local informal opinion leader who has received training from the study U/S lead (JC). The goals of the training session will be to: i) **interactively discuss** the rationale of regional anesthesia in hip fractures. Opinion leaders will use narrative case presentations to illustrate the ***known relative advantage*** of using USGRA for patients (instant pain control), for the ED physician (financial incentives) and the health care system (reduced care burden and potential to reduce delirium); ii) **teach ED physicians how to perform ultrasound-guided regional anesthesia**. A detailed description of the procedure is provided in Appendix 1. In summary, the technique involves 1) identifying the femoral nerve, artery, vein, and the Fascia Iliaca plane using ultrasound. Both needle and fascial plane are bright white structures on the ultrasound. 2) A needle is introduced just lateral to the probe and advanced towards the femoral nerve until the needle crosses the Fascia Iliaca. Bupivicaine, 20 cc's, is then injected under direct visualization adjacent to the femoral nerve, improving block effectiveness and safety. Our pilot testing confirmed that all participants could acquire this skill, and trained physicians subsequently used the block in 80% of hip fractures during the pilot study.[100]

Our tested 2 hour session to train physicians to perform USGRA will include (a) 30 minutes to interactively present the relevant anatomy, safety and practical considerations; (b) a 90 minute hands-on training session with 3 stations.

The 3 stations include:
i) A "blue phantom" station using a gel-block simulation model that is an ultrasonographically accurate representation of the anatomy of the femoral neurovascular structures. Physicians use this model to consolidate their sonoanatomy, as well as to practice the hand-eye coordination skills needed to locate the relevant landmarks with the ultrasound machine.

ii) We developed a novel turkey thigh model station during our pilot work to allow physicians to practice the manual skill of passing a needle under ultrasound guidance and practice the injection of local anesthetic into a specific fascial plane.

iii) A live human model station that allows physicians to run thorough the procedural steps and integrate their sonoanatomy in a human model. Note that no actual needle punctures are used in this station. Physician competence is assessed using a check list assessment during this last station.

3. Establishing Local Targets and Committing to Achieve Them. Local achievable targets for the time to providing analgesic control using regional analgesia or standard of care opioid analgesics for hip fracture patients will be established in consultation with each ED group, up to a maximum of 120 minutes from radiologic confirmation of the fracture. Once targets are agreed upon at each site, participating physicians will be asked to commit to achieving those targets 85% of the time.
4. Coaching. Any intervention physician that identifies a lack of comfort with the USGRA after training will be offered the option of having a "coach" physician work on the same shift as them. The coach will have experience in the use of the USGRA and will assist intervention physicians in successfully achieving their first USGRA. Note, although reasonable efforts will be made, a coach will be scheduled only if this is practical to arrange at each site in keeping with our pragmatic trial design.
5. Audit & Timely Feedback. Feedback on enrolled and missed eligible patients and protocol violations will be provided to intervention physicians within 72 hours by the local Research Coordinator. In addition, qualitative feedback from patients as well as clinical updates will be collected

by the Research Coordinator and relayed to the treating intervention physicians on a monthly basis and monthly tallies of the study enrollment will be provided to intervention physicians.
6. Reminders. Methods for reminding intervention physicians of the study will be chosen by each ED group and adapted to the local environment from a list provided by the Research Coordinator. Examples include posters, screen savers and e-mails from the Research Coordinator before each ED shift or automated notifications linked to clinical information systems where feasible.
7. Incentives. Providing regional anesthesia is a remunerable procedure under the provincial health insurance plans in both Ontario and Quebec. In addition, participating physicians will receive training by the end of the trial to promote participation, with a maximum delay of 18 months. Our pilot study revealed that over 92.4% of respondents were willing to participate using this strategy.
Control group. All physicians will start in the control group until they are trained and cross over to the intervention group - i.e. they will provide the **current local standard of analgesic care** for hip fracture patients such as the use of IV opioids with supplemental acetaminophen and non-steroidal anti-inflammatory agents. ED physicians in both groups will be encouraged to provide pain control within 120 minutes of diagnosis.

2.2 Allocation of Participants, Randomization and Justification and Description of Clusters

All eligible patients seen by a participating ED physician during the study period will form a cluster. We will adopt clear rules to attribute hip fracture patients to a specific ED physician to limit the potential for selection bias. All participating ED's work in a major care zone, with one staff physician identified as being responsible for that zone. Hip fractures are typically treated in the major care zone, since they are easily identified at triage, and are unable to ambulate. To minimize selection bias due to selective delays to see a patient, any patient who has an X-Ray completed more than 60 minutes prior to the end of the shift will be assigned to that physician and analyzed on an **intention to treat basis.** Similarly, patients who do not receive their assigned intervention will be included in the study, and will also be analyzed according to the intention to treat principle.

All participating EDs employ a fixed template to schedule ED physicians. For example, at our pilot site, Sunnybrook, there are 17 full time equivalent shift lines that cover all available shifts. All 17 shifts lines are identical repeating pattern that start at different points in the template. This ensures an equitable distribution of shifts among all members over the full 17 week schedule.

2.3 Protection against Bias

2.3.1 Blinding: We do not believe blinding of the physician and patient with a sham injection in the control group is ethically justifiable. Instead, a **blinded adjudication panel** of 3 clinicians (ED Physician, Geriatrician and psychologist or psychiatrist) will make the final determination of the primary outcome, delirium. Adjudicators will have access to all study documentation, chart material, and transcripts of family interviews and will use a validated delirium assessment tool,[110,111] but will have no contact with study patients, and all documentation will be anonymized and blinded as to the randomization status of patients. Finally, the study statistician will remain blinded to the intervention status of physicians until after the analysis is completed.

2.3.2 Prevention of cross over: Some knowledge can easily be transferred between participants at a site (e.g. knowledge that one antibiotic is more effective). In contrast, U/S guided RA is a manual skill. Previous literature, confirmed in our pilot training, demonstrates that this is a skill that ED physicians can readily acquire, but requires 2 hours of hands-on training. Participants also found that use of a mannequin simulator was important before trainees felt confident in identifying landmarks on U/S, and only research staff will not have access to this mannequin simulator.

To further minimize cross-over, the nature of the proposed randomized trial was discussed with ED physicians at participating sites and a consensus was reached that ED physicians should receive adequately training before attempting to use U/S guided RA, and the importance of following randomization for the integrity of the research project was discussed. Our pilot survey revealed that 92.4% of ED physicians stated they would respect their randomization group for the duration of the trial. This was re-enforced by the Department Chiefs at all participating sites (see Letters of Support).

We then tested if these protections against cross-over would be effective. First, we monitored the use of U/S guided RA at Sunnybrook since the initiation of our pilot work using focus groups and chart reviews of all hip fractures seen. Despite the fact that our pilot work has raised awareness of this procedure, we found no spontaneous uptake of U/S guided RA by untrained physicians. However, we realize that we may have missed the undocumented use of U/S guided RA. Therefore, we conducted a 14 month prospective pilot study during which all hip fracture patients were prospectively interviewed by a research assistant. Of 67 hip fractures seen over 14 months, only one control patient received U/S guided RA by an off-service fellow with previous training. In no case did a trained physician introduce the procedure to an untrained physician.

Despite these protections, we cannot guarantee that no cross-over contamination will occur. However, given an intention to treat analysis, bias due to cross-over is likely to be conservative, biasing the study results towards the null hypothesis. Thus, we would have confidence that any statistically significant improvement in delirium in the intervention groups was valid and not due to cross-over contamination.

2.4 Inclusion / Exclusion Criteria There are inclusion and exclusion criteria at the cluster and individual patient level. At the cluster level, ED physicians practicing at a participating site will be eligible. Exclusion criteria include ED physicians who work casually (less than 0.25 FTE), are routinely using U/S guided RA for hip fracture patients, or decline participation in the trial. At the patient level, all hip fractures seen by a participating ED physician will be eligible. Patient level exclusion criteria include: age less than 65 years; delirious on initial assessment by ED physician or severe dementia, (see Appendix 16 for details); communication problems (critically ill, unconscious, language barrier despite use of secure telephone-based translation services; allergies to narcotics or local anesthetic; or anticoagulant use (e.g. warfarin, dabigatran, rivaroxaban). ED physicians will be trained to assess delirium using the CAM at the start of the study. Patients with hip fractures not requiring surgery (e.g. greater trochanter avulsion) will also be excluded.

2.5 Duration of Treatment Period The objective of this pragmatic clinical trial is to assess whether the administration of a single pre-operative nerve block with 20 cc of bupivicaine reduces subsequent delirium. The duration of the block will be variable, but previous studies have shown that this is sufficient to reduce delirium.[11] Pain management will otherwise be administered according to the standard of care at each of the participating centers in both intervention and control groups.

2.6 Follow-up Duration / Frequency For the primary outcome, time to onset of post-injury delirium, previous studies have shown that 86% of patients develop delirium within the first 3 days.[112] We will continue daily assessment for the primary outcome until 7 days post-injury. Hip fracture patients are routinely transferred to a rehabilitation facility at day 4-7 post-injury, but will be contacted by a Research Assistant until 7 days post-injury to conduct the Telephone Interview of Cognitive Status (TICS) if they have been transferred prior to 7 days post-injury ,[113] a validated telephone screening method for detecting delirium. Any suspected cases of delirium will be confirmed by face-to-face administration of the CAM. In addition, we will obtain 1 month and 1 year telephone follow-up on patients to assess cognition and functional status. Patients with suspected changes in mental status will also have face-to-face interviews.

2.7 Outcome Measures

2.8.1 Primary Outcome:

Our primary outcome is the time to onset of incident delirium as well as overall rate of incident delirium among patients treated by participating ED physicians. Delirium will be measured daily using the Confusion Assessment Method (CAM) supplemented by use of a validated chart review tool (See Appendix 7 and Appendix 8). [110,111,114] Because mental status fluctuates during delirium, the CAM user manual specifies that clinical staff and family members should be interviewed and clinical notes reviewed to determine if there is evidence of delirium since the previous interview. This method has been previously validated in post-operative patients and extensively used to measure delirium post hip fracture repair.[11,55,61,112,115-120] The use of the CAM in the ED has also been validated and was shown to have a high degree of sensitivity (86%) and specificity of (100%).[121,122] A recent review of 4000 studies

that used the CAM to measure delirium found sensitivities across settings of 94% and specificities of 89%.[123]

2.8.2 Secondary Outcome Measures: We will measure additional outcomes in 4 domains.

<u>I Uptake of USGRA</u>: 1) The rate of USGRA use by trained physicians will be our main secondary outcome. Our target is to achieve 70% compliance. **2)** Time to perform the block will be recorded by the ED physician. The central Project Coordinator will conduct quarterly plus end of study interviews with site coordinators to assess any site-specific differences in application of the KTP strategies. **<u>II Pain Control</u>**: 3) Pain severity, recorded by the ED physician using the 10 point numeric rating scale,[124] on initial assessment and at20 to 30 minutes after administration of regional anesthesia or opioid analgesia, and then assessed daily by the RA to day 7 post-injury. **4)** Use of supplementary analgesics, measured by total dose of opioid in morphine equivalent units up to day 7. **5)** ED physician perception of block effectiveness on a 7 point Likert scale.

<u>III Other Delirium Measures:</u> 6) Maximum severity of delirium using the validated Delirium Index (DI) measured daily by research assistants up to day 7 post-injury.[125] **7)** Time to delirium onset, measured from ED triage to time of first positive CAM. **<u>IV Adverse Events:</u> 8)** Complications associated with USGRA including local hematoma and damage to nervous structure, assessed by the anesthetist peri-operatively. **8)** In-hospital falls and death, as assessed during patient interview and chart review. **9)** Cardiovascular events, specifically myocardial infarction using WHO criteria.

2.8 <u>Outcome Measurement at Follow-up:</u> Delirium assessments will occur every 24 hours for the first 7 post-injury days. Assessment of health care use, functional status, cognitive status and living situation will be conducted by telephone follow-up at 1 month and 1 year.

2.9 <u>Health Services Research Issues</u>: Health Services Outcomes to be measured include: **10)** Hospital length of stay, measured from time of triage in the ED to time of discharge or death. **11)** Functional status before hip fracture, 1 month and 1 year, measured using the validated Older American Resources and Services (OARS) activities of daily living (ADL) questionnaire.[126] **12)** Return to independent living at discharge, will be assessed by a telephone interview 1 month and 1 year after discharge. **13)** Mortality, return to hospital or return to ED within 1 year, as assessed by telephone interviews, verified against patient records, at 1 month and 1 year. **14)** Quality of life measures including the SF-12will be assessed by telephone interview at 1 month and 1 year. **15)** We will compare total direct costs of care for intervention and control subjects based on immediate and downstream hospital LOS and intensity of care (e.g. transfer to ICU). Costs of training for physicians will be included in sensitivity analyses.[127-129]

2.10 <u>Sample Size Calculation & Feasibility:</u>

Sample size is based on the primary outcome, the rate of incident delirium. Our pilot surveys found 92.4% of physicians were willing to participate. To be conservative, we assumed a participation rate of 80% of the 204 eligible physicians (MDs) or 160. We will use a stepped-wedged design. Rather than training half of MDs at the beginning of the study, we will train them in groups of 4-8 at 4-12 week intervals, using untrained MDs as the control group. We expect each MD to treat 5- 6 patients. Assuming an ICC of 0.01 to 0.04, at the point at which half of the physicians have been trained (e.g. 72 trained, 72 untrained), this sample size will provide 80% power at alpha of 0.05 to detect a difference in delirium rates of 7.6% to 8.0 %. The following table shows that under a variety of scenarios, given a sample size of 360 to 420 patients per group (trained, untrained physicians), our study will have 80% power to detect a clinical significant difference in delirium rates of 7.0 to 8.0%.

	Group 1 Clusters/ Patients	Total Patients, Group 1	Group 2 Clusters/ Patients	Total Patients, Group 2	Intra Cluster Corr	Prop Grp 2 Control	Prop Grp 1 or |H0 Trtmnt	Prop|H1 Grp 1 or H1 Trtmnt	Diff if H0	Diff if H1
**Power**	**K1/M1**	**n**	**K2/M2**	**n**	**ICC**	**P2**	**P1.0**	**P1.1**	**D0**	**D1**
0.80	72/5	360	72/5	360	0.010	0.2100	0.2100	0.1305	0.0000	-0.0795
0.80	72/6	432	72/6	432	0.020	0.2100	0.2100	0.1349	0.0000	-0.0751

0.80	72/6	432	72/6	432	0.030	0.2100	0.2100	0.1333	0.0000	-0.0767
0.80	84/5	420	84/5	420	0.020	0.2100	0.2100	0.1345	0.0000	-0.0755
0.80	84/5	420	84/5	420	0.030	0.2100	0.2100	0.1333	0.0000	-0.0767
0.80	84/6	504	84/6	504	0.040	0.2100	0.2100	0.1371	0.0000	-0.0729

2.11 Planned Recruitment Rate: Eligible patients
Volumes and waiting times for hip fracture repair are closely monitored in Ontario, and data collection is standardized and subject to quality audits by the Canadian Institutes for Health Information (CIHI). The most recently available CIHI data from the last 2 quarters for fiscal year 2010-2011 showed that there were 1313 hip fractures/year at the 6 participating centers (see Appendix 10). Our pilot data showed that 41.7% of patients were ineligible due to dementia, language barriers, or anticoagulant use. A further 12.1% declined to participate leaving 46.2% of the 1313 patients, or 606 eligible subjects / year. Thus we will reach our total sample size or 800 subjects in 18 to 20 months.

2.12 Compliance – Anticipated Problems and Justification of Compliance figures
There are two threats to compliance in our proposed trial. First, physicians may attempt to perform the block prior to receiving training (early cross over). As addressed in section 2.4.2, the USGRA procedure cannot easily be transferred, and the chiefs of all participating ED's have agreed that physicians will not use the procedure unless they are trained by the research team (see attached letters of support). Further, our pilot data showed only one case of cross-over contamination in 14 months, and that happened when an off-service fellow performed the block. The second type of potential "non-compliance" occurs when a trained physicians fails to administer the block to hip fracture patients. Given that we are testing a knowledge-to-practice intervention, this is a key study outcome and section 2.2.1 outlines all the measures we have taken to promote uptake of USGRA. We tested this strategy in a pilot study at Sunnybrook where we randomly assigned 13 ED physicians to the KTP intervention. These physicians saw 24 eligible hip fracture patients, 20/24 received the block (83.3%, 95%CI 62.6 to 95.3%). But the central project coordinator will actively engage with site coordinators and closely monitor any local barriers that impede USGRA use at other sites (e.g. availability of study materials and block kits). Barriers, facilitators and local issues will be recorded to guide future implementation and dissemination.

2.13 Lost to Follow-up rate and justification. To minimize loss to follow-up, research assistants will establish the anticipated discharge date for study patients on the first post-injury day and will verify any planned changes with clinical staff daily. For health services outcomes including death within one year and subsequent hospitalization, individual patient data will be contacted by telephone at 1 and 12 months. Our 14 month pilot study revealed a loss-to-follow-up rate of 1/67 for our primary outcome, delirium at post-injury day 7. Although we anticipate a lost-to-follow-up of less than 10%, we will be able to complete the trial within 4 years even if the lost-to-follow-up rate is 20%.

2.14 Settings and Number of Centers. We have recruited six academic teaching hospitals: Kingston General Hospital, Mount Sinai Hospital, the Ottawa Hospital (Civic and General sites), l'Hôpital Enfant-Jésus in Québec City and l'Hôpital Sacré-Coeur de Montréal. All sites have extensive history participating in multicenter studies.

2.15 Proposed Analysis An intention-to-treat analysis will be used. For the primary outcome, the proportion of patients with any delirium in the intervention group will be compared to the control group using a generalized estimating equation (GEE) model with a logit link function adjusting for the correlation among observations taken by the same physician. The estimates from this model will also be compared to a generalized linear mixed model (GLMM) which will include a random term to account for baseline physician differences, [131] as the potential impact of physicians on baseline delirium rate is unknown. While we expect randomization to balance potential confounders associated with delirium

between the two groups, we will assess for imbalances between groups that arose despite randomization for the following important confounders identified a-priori from existing systematic literature reviews: Age, gender baseline dementia, visual or hearing impairment, residence in a nursing home, time in the ED time and time to surgery. If any confounders differ between groups by more than a clinically significant threshold identified a-priori, (see Appendix 12), they will be adjusted in the GEE model. Between-group comparisons are planned for the following categorical secondary outcomes using logistic regression: Deaths, cardiovascular events, in-hospital falls, patients who return to independent living and delirium at discharge. The following continuous secondary outcomes will be compared using GLM or survival analysis: Delirium severity, time to delirium, hospital length of stay and functional status. For remaining secondary outcomes, 95% confidence intervals will be reported as appropriate.

2.16 Proposed Frequency of Analysis: No interim analysis will be carried out

2.17 Planned Sub-group Analysis:

One previous RCT found that RA in hip fractures effectively reduced delirium in patients at moderate risk for delirium, but not among high-risk patients. Therefore, we will measure the same Inouye Delirium Risk assessment scale used in the previous RCT to conduct a sub-group analysis. As well, we will examine gender and time from admission to surgery in sub-group analyses to assess the potential impact of these confounders.

2.18 Pilot study of design: Please see 1.39 for details.

3.0 TRIAL MANAGEMENT

3.1 Day to day management. Trial recruitment will use a secure web-based application that we have extensively used in previous studies and piloted in 67 hip fracture patients at Sunnybrook. ED physicians identify patients with hip fracture using a simple web-based enrollment form. The form assists the ED Physician to confirm all eligibility criteria, capture pain scores, patients' initial delirium status and notifies the Research Coordinator (RC) that an eligible patient has been assessed. The RC's at each site will follow patients within 24 hours, obtain informed consent, and start data collection. In addition, the central site coordinator can access online enrollment statistics. We have a long history of working with our partner sites, and have used a similar system to monitor patient enrollment accurately.

3.2 Role of Principal Applicants and Co-Applicants.

We have assembled a team of investigators with a wide range of skills and experience that is passionately committed to creating new knowledge that can quickly lead to improved patient care. Because of the size of our team please see Appendix 14 for a complete list of our applicants and opinion leaders. Dr. Jacques Lee, principal applicant, is an ED physician and scientist focused on improving the care of older ED patients, and is responsible for overall conduct of the trial. He is PI on 3 CIHR grants and is experienced in the design, implementation and analysis of multi-site projects[105,132-143] He has been PI of a previous RCT[144] and has experience as a co-author and site coordinator for ED Pharma trials.[145] Dr. Jordan Chenkin is the U/S lead at Sunnybrook, and is experienced in the design, delivery and evaluation of courses to improve U/S skills, and has collaborated with MW to develop and test our training. Dr. Howard Ovens is the Director of the Schwartz /Reisman Emergency Centre at Mt. Sinai for the past 12 years and is the ED Lead for the TCLHIN and is excellently placed to disseminate our results broadly. Dr. Anil Chopra is the Division Head of Emergency Medicine at University of Toronto, has a health services research interest, will be the project lead at UHN, and has developed a mature U/S program at UHN. Dr. Marco Sivilotti is the chair of the Emergency Medicine research committee at Queens University, and will assist Dr Joseph Newbigging in trial supervision at KGH. Dr. Hans Kreder is chair of Orthopedic Clinical Epidemiology at Sunnybrook, Chief of the Musculoskeletal program, and Chair of the Canadian Orthopedic Association. He will assist in data analysis and interpretation. Given his national profile, he is excellent placed to disseminate the results of this trial and to influence decision makers. Dr. Jeffrey Perry is a

Senior Scientist at the Ottawa Hospital Research Institute. He will be the site investigator in Ottawa, responsible for oversight of the trial. Dr. Michael Woo is the Director of ED U/S at the University of Ottawa and is the local opinion leader and will provide local coaching. Dr. Alexander Kiss, the study biostatistician, has extensive experience with our team in study design & analysis, and will primarily be responsible for the statistical analysis. Dr. David Ryan is the director of Knowledge Translation for the Toronto Regional Geriatrics Program. He is primarily responsible for the analysis, design and oversight of KTP strategies, and monitoring of KTP implementation. Dr. Raoul Daoust (Clinical Associate Professor, researcher), our newest team member, is an accomplished ED pain researcher and will lead the study at Hôpital Sacré-Cœur de Montréal. Dr. Marcel Émond is the Principal Investigator of the Canadian Emergency department Team Initiative (CETI), a CIHR supported Emerging Team grant. CETI has recruited over 1500 elders in 10 Canadian EDs. He will lead and supervise the recruitment in Québec.

3.3 Trial Steering Committee / Data Monitoring / Safety Committee

USGRA has a well-established safety profile.[9] Nonetheless, a safety committee (1 ED physician, 1 Anesthetist, 1 Biostatistician) will review all adverse outcomes at midpoint in the trial, and employ a stopping rule if serious adverse event rate exceed 10% absolute.
