# Supplementary material for: Ultrasound-Guided Regional Anesthesia by Emergency Physicians for Hip Fractures and Delirium: A Randomized Clinical Trial
Source: JAMA Netw Open. 2025 Dec 15;8(12):e2549337. doi: 10.1001/jamanetworkopen.2025.49337 (PMC12706686; doi:10.1001/jamanetworkopen.2025.49337)
Supplement: Supplement 2. — eFigure. CONSORT Modified Stepped Wedge Randomized Clinical Trial Flow Diagram [file jamanetwopen-e2549337-s002.pdf]

Supplemental Online Content

Lee JS, Chenkin J, Simard R, et al. Ultrasound-guided regional anesthesia by emergency physicians for hip fractures and delirium: a randomized clinical trial. *JAMA Netw Open*. 2025;8(12):e2549337. doi:10.1001/jamanetworkopen.2025.49337

eFigure. CONSORT Modified Stepped Wedge Randomized Clinical Trial Flow Diagram

This supplemental material has been provided by the authors to give readers additional information about their work.

© 2025 Lee JS et al. *JAMA Network Open*.

CONSORT Modified Stepped Wedge Randomized Clinical Trial Flow Diagram

Date	Site 1	Delirium	Site 2	Delirium	Site 3	Delirium	Site 4	Delirium	Site 5	Delirium	*
Jun-2016					MDs Trained: 1						
					Cont: 0	-					
					Inter: 1	0					
Dec-2016			MDs Trained: 3						MDs Trained: 1		
			Cont: 3	1					Cont: 0	-	
			Inter: 23	7					Inter: 1	0	
Jan-2017			MDs Trained: 1								
			Cont: 1	1							
			Inter: 13	3							
Feb-2017	MDs Trained: 2		MDs Trained: 2		MDs Trained: 2		MDs Trained: 3		MDs Trained: 1		
	Cont: 0	-	Cont: 2	0	Cont: 0	-	Cont: 1	0	Cont: 0	-	
	Inter: 3		Inter: 28	8	Inter: 4	0	Inter: 5	1	Inter: 3	2	
Mar-2017			MDs Trained: 1				MDs Trained: 4		MDs Trained: 5		
			Cont: 0	0			Cont: 0	-	Cont: 0	-	
			Inter: 8	1			Inter: 9	3	Inter: 10	3	
Apr-2017	MDs Trained: 2				MDs Trained: 1		MDs Trained: 2		MDs Trained: 2		
	Cont: 4				Cont: 1	0	Cont: 2	2	Cont: 0	-	
	Inter: 6				Inter: 2	0	Inter: 2	2	Inter: 3	1	
May-2017			MDs Trained: 3		MDs Trained: 2		MDs Trained: 3		MDs Trained: 2		
			Cont: 2	2	Cont: 1	0	Cont: 3	0	Cont: 0	-	
			Inter: 23	5	Inter:15	1	Inter: 5	1	Inter: 3	2	
Jun-2017	MDs Trained: 2				MDs Trained: 3		MDs Trained: 2		MDs Trained: 4		
	Cont: 0	-			Cont: 2	0	Cont: 2	1	Cont: 0	-	
	Inter: 5				Inter: 4	0	Inter: 4	2	Inter: 12	4	
Jul-2017			MDs Trained: 2				MDs Trained: 2		MDs Trained: 4		
			Cont: 9	3			Cont: 1	1	Cont: 0	-	
			Inter: 13	2			Inter: 9	4	Inter: 3	1	
Aug-2017									MDs Trained: 1		
									Cont: 0	-	
									Inter: 1	0	
Sep-2017	MDs Trained: 1		MDs Trained: 1		MDs Trained: 3		MDs Trained: 3		MDs Trained: 3		
	Cont: 0	-	Cont: 2	0	Cont: 2	0	Cont: 2	0	Cont: 1	1	
	Inter: 1		Inter: 2	0	Inter: 5	0	Inter: 3	0	Inter: 3	0	
Oct-2017			MDs Trained: 3		MDs Trained: 2						
			Cont: 9	5	Cont: 1	0					
			Inter: 17	3	Inter: 1	0					

Date	Site 6	Delirium	Site 7	Delirium
Jan-2018	MDs Trained: 1			
	Cont: 0	-		
	Inter: 4	0		
Feb-2018			MDs Trained: 1	
			Cont: 2	1
			Inter: 3	0
Mar-2018			MDs Trained: 2	
			Cont: 4	1
			Inter: 18	4
Apr-2018				
May-2018			MDs Trained: 3	
			Cont: 6	2
			Inter: 14	4
Jun-2018			MDs Trained: 3	
			14	0
			Inter: 11	0
Jul-2018				
Aug-2018				
Sep-2018			MDs Trained: 4	
			Cont: 14	2
			Inter: 9	2
Oct-2018	MDs Trained: 5		MDs Trained: 2	
	Cont: 4	1	Cont: 5	2
	Inter: 3	0	Inter: 6	2
Nov-2018	MDs Trained: 2		MDs Trained: 1	
	Cont: 2	0	Cont: 4	3
	Inter: 0	-	Inter: 0	-
Dec-2018			MDs Trained: 1	
			Cont: 4	3
			Inter: 0	-

© 2025 Lee JS et al. *JAMA Network Open*.

Date	Site 1	Delirium	Site 2	Delirium	Site 3	Delirium	Site 4	Delirium	Site 5	Delirium
Nov-2017					MDs Trained: 2		MDs Trained: 4		MDs Trained: 4	
					Cont: 1	0	Cont: 4	0	Cont: 5	3
					Inter: 2	0	Inter: 5	2	Inter: 3	1
Dec-2017	MDs Trained: 1		MDs Trained: 3				MDs Trained: 4		MDs Trained: 4	
	Cont: 0		Cont: 14	4			Cont: 3	2	Cont: 4	3
	Inter: 1		Inter: 19	5			Inter: 7	3	Inter: 5	1
Jan-2018							MDs Trained: 1		MDs Trained: 2	
							Cont: 0	-	Cont: 3	2
							Inter: 1	1	Inter: 2	0
Feb-2018	MDs Trained: 1		MDs Trained: 2		MDs Trained: 3		MDs Trained: 1		MDs Trained: 2	
	Cont: 1		Cont: 4	1	Cont: 4	0	Cont: 5	0	Cont: 2	1
	Inter: 0		Inter: 3	1	Inter: 3	0	Inter: 2	1	Inter: 6	3
Mar-2018							MDs Trained: 3		MDs Trained: 1	
							Cont: 3	1	Cont: 0	-
							Inter: 3	0	Inter: 1	0
Apr-2018	MDs Trained: 1				MDs Trained: 3		MDs Trained: 2		MDs Trained: 7	
	Cont: 0				Cont: 5	0	Cont: 8	2	Cont: 8	2
	Inter: 1				Inter: 2	0	Inter: 6	1	Inter: 8	4
May-2018							MDs Trained: 6		MDs Trained: 2	
							Cont: 6	4	Cont: 2	1
							Inter: 2	0	Inter: 1	0
Jun-2018					MDs Trained: 3					
					Cont: 2	0				
					Inter: 1	0				
Jul-2018	MDs Trained: 1									
	Cont: 2									
	Inter: 0									
Aug-2018							MDs Trained: 1			
							Cont: 1	1		
							Inter: 0	-		
Sep-2018										
Oct-2018										
Nov-2018			MDs Trained: 1		MDs Trained: 1					
			Cont: 4	1	Cont: 3	0				
			Inter: 9	1	Inter: 1	0				

*

Date	Site 6	Delirium	Site 7	Delirium
Jan-2019			MDs Trained: 3	
			Cont: 17	5
			Inter: 7	3
Feb-2019	MDs Trained: 6			
	Cont: 11	5		
	Inter: 0	0		
Mar-2019			MDs Trained: 3	
			Cont: 7	2
			Inter: 15	4
Apr-2019	MDs Trained: 6			
	Cont: 5	3		
	Inter: 3	0		
May-2019	MDs Trained: 5			
	Cont: 7	1		
	Inter: 3	0		
Jun-2019	MDs Trained: 2			
	Cont: 2	0		
	Inter: 0	-		

© 2025 Lee JS et al. *JAMA Network Open*.

Supplementary Figure

CONSORT Modified Stepped Wedge Randomized Clinical Trial Flow Diagram

The number of physicians trained each month, the number of control and intervention patients treated by the physicians, and the number who developed delirium are presented.

Cont = Control Group
Inter = Intervention Group
MDs = Physicians

* Note, sites 6 & 7 started training session in January 2018

© 2025 Lee JS et al. *JAMA Network Open*.
