# Supplementary material for: Ultrasound-Guided Regional Anesthesia by Emergency Physicians for Hip Fractures and Delirium: A Randomized Clinical Trial
Source: JAMA Netw Open. 2025 Dec 15;8(12):e2549337. doi: 10.1001/jamanetworkopen.2025.49337 (PMC12706686; doi:10.1001/jamanetworkopen.2025.49337)
Supplement: Supplement 3. — Data Sharing Statement [file jamanetwopen-e2549337-s003.pdf]

Data Sharing Statement

Lee. Ultrasound-Guided Regional Anesthesia by Emergency Physicians for Hip Fractures and Delirium: A Randomized Clinical Trial. *JAMA Netw Open*. Published online December 15, 2025. doi:10.1001/jamanetworkopen.2025.49337

Data
Additional Information: Organization - Clinicaltrials.gov Trial Number: NCT02892968 URL: clinicaltrials.gov/study/NCT02892968
Data available: Yes
Data types: Other (please specify)
Additional Information: Deidentified data will be made avaialable on reasonable request
How to access data: jacques.lee@sinaihealth.ca
When available: With publication

Supporting Documents
Document types: None

Additional Information
Who can access the data: NA
Types of analyses: NA
Mechanisms of data availability: NA
